# Identification of factors related to immunotherapy efficacy and prognosis in patients with advanced head and neck squamous cell carcinoma

**DOI:** 10.1186/s13000-021-01147-7

**Published:** 2021-11-25

**Authors:** Xuanli Xu, Rongrong Li, Lin Zhang, Guopei Zhu, Dandan Ren, Lijia Wu, Xiaoli Gong

**Affiliations:** 1 Department of Oral and Maxillofacial Head and Neck Oncology, the Ninth People’s Hospital Affiliated to Shanghai Jiaotong University School of Medicine, No.639 Zhizaoju Road, Huangpu District, Shanghai, 200011 China; 2 Genecast Biotechnology Co., Ltd, 88 Danshan Road, Xidong Chuangrong Building 214104 Wuxi, China

**Keywords:** Head and neck squamous cell carcinoma, Immunotherapy, Prognosis, Efficacy

## Abstract

**Background:**

Immunotherapy is an important treatment in oncology, but only a fraction of patients with head and neck squamous cell carcinoma (HNSCC) benefit from it. Therefore, the aim of this study was to identify predictive biomarkers of immunotherapy response for HNSCC in order to improve treatment outcomes.

**Methods:**

Survival analyses and comparative efficacy evaluation were performed to investigate prognostic and therapeutic impact factors in patients with advanced HNSCC following immunotherapy, and to examine the effects of factors including gene mutations, tumor mutational burden (TMB), mutant-allele tumor heterogeneity (MATH), and immune cell infiltration on the survival and efficacy.

**Results:**

Anti-PD-1 treatment led to a prolonged overall survival (OS) in HNSCC patients with gene mutations compared with those without the mutations, while no significant difference in the OS was found between the two groups of patients. And no marked association between the MATH value and OS was detected in HNSCC patients, whereas patients with either high TMB scores in tissues and blood or high immune cell infiltration displayed a significantly longer OS. Further analysis with efficacy as the primary endpoint revealed no significant differences in the tissue TMB, blood TMB, and MATH value between the patients who responded to immunotherapy and those who did not. Moreover, no significant differences in the expression percentages of positive immune cells in tumor, stroma, and total regions were identified between the above two groups of patients.

**Conclusion:**

HNSCC is characterized by high mutation rate, high mutation burden, and high level of immune cell infiltration, and a subset of HNSCC patients respond to immunotherapy. Here, we showed that high mutation burden and immune cell infiltration may improve the prognosis of HNSCC patients with immunotherapy, while there was no remarkable effect on the efficacy.

**Supplementary Information:**

The online version contains supplementary material available at 10.1186/s13000-021-01147-7.

## Background

Head and neck squamous cell carcinoma (HNSCC) is one of the most common malignant tumors worldwide, with 1.45 million new cases and 500,000 deaths each year [1]. Comprehensive treatment methods such as surgery, chemoradiotherapy, and molecular targeting are commonly used in the treatment of locally advanced patients [2]. However, these treatment strategies are associated with severe acute and long-term toxicity, and more than half of patients eventually developed cancer recurrence or distant metastasis. For patients with recurrence or distant metastasis, treatment options are particularly limited, and the prognosis is poor; the median overall survival (OS) after diagnosis is less than 1 year [3].

In recent years, in-depth studies have advanced our understanding of the complex connection between HNSCC and the immune system, as well as the various mechanisms by which HNSCC escapes immune surveillance. Immunotherapy is based on immune escape mechanism, and has shown promising prospects in tumor treatment, especially in treating HNSCC patients with recurrence and metastasis; the curative effect of immunotherapy cannot be achieved by traditional therapy [4]. Based on transcriptome data from the Cancer Genome Atlas, characterization of the immune status of HNSCC revealed a prominent immune infiltration in the tumors with the highest levels of CD8+ T cells and activated NK cells, as well as the marked expression of regulatory T cells and related immune checkpoints including PD-1, CTLA-4, GITR, ICOS, and IDO [5]. HNSCC has strong immunogenic characteristics, while alleviation of cancer-potentiated immune suppression has demonstrated the potential to reduce the tumor burden of HNSCC and to improve the quality of life of patients [6, 7]. Despite the huge progresses in the treatment of patients with advanced HNSCC made by immunotherapy, only a small proportion of patients have benefited from this therapy [8]. Thus, it is of great significance to screen and identify the relevant biomarkers of immunotherapy efficacy and prognosis of HNSCC patients.

In this study, the clinical data of 44 patients with advanced HNSCC treated with immunotherapy were retrospectively analyzed to identify related factors affecting the effect and prognosis of HNSCC treatment.

## Material and methods

### Patients

This study included a total of 44 histologically diagnosed HNSCC patients who received immunotherapy (PD1), staged and graded according to the 7th edition of the American Joint Committee on Cancer, and tumor specimens and matched blood samples were collected [9]. 10 mL of whole blood was collected from the patients in a heparin-coated tube. Tumor tissue DNA and plasma cfDNA were extracted respectively. However, efficacy evaluation was available for only 37 patients. The tumor specimens taken from patients with curative effect appraisal data (*n* = 37) were analyzed for assessing therapeutic efficacy, and patients were divided into the response group (*n* = 29) and non-response group (*n* = 8) based on whether they responded to PD1 treatment (Table 1 and Table S1). Targeted NGS panels were analyzed to analyze the genomic profile of tumors and plasma samples, and 5 tumor specimens were excluded by data quality control. Finally, 33 patients had both efficacy assessments and tumor DNA data available (Figure S1). All patients provided written informed consent. This study was approved by the Ninth People’s Hospital Affiliated to Shanghai Jiaotong University School of Medicine.

**Table 1:** The clinical features between response and nonresponse groups in the HNSC patients

Features	Overall (*n* = 37)	Response (n = 29)	Nonresponse (n = 8)	***p***-value
**Gender** (%)				1.000
Male	31 (83.8)	24 (82.8)	7 (87.5)	
Female	6 (16.2)	5 (17.2)	1 (12.5)	
**Age** (years)				0.941
median	57.0	57.0	56.5	
IQR	50.0–64.0	49.0–64.0	53.8–63.8	
**Primary site** (%)				0.631
Oral cavity	30 (81.1)	24 (82.8)	6 (75.0)	
Oropharynx	7 (18.9)	5 (17.2)	2 (25.0)	
**HPV status (%)**				
Negative	7 (18.9)	5 (17.2)	2 (25.0)	NA
NA	30 (81.1)	24 (82.8)	6 (75.0)	
**p Stage** (%)				1.000
IVa	26 (70.3)	19 (65.5)	7 (87.5)	
IVb	2 (5.4)	2 (6.9)	0 (0.0)	
IVc	6 (16.2)	5 (17.2)	1 (12.5)	
NA	3 (8.1)	3 (10.3)	0 (0.0)	
**T stage** (%)				0.099
T1	1 (2.7)	1 (3.4)	0 (0.0)	
T2	4 (10.8)	2 (6.9)	2 (25.0)	
T4	19 (51.4)	18 (62.1)	1 (12.5)	
NA	13 (35.1)	8 (27.6)	5 (62.5)	
**N stage** (%)				0.664
N1	3 (8.1)	3 (10.3)	0 (0.0)	
N2	20 (54.1)	13 (44.8)	7 (87.5)	
N3	1 (2.7)	1 (3.4)	0 (0.0)	
NA	13 (35.1)	12 (41.4)	1 (12.5)	
**Tobacco** (%)				0.670
Yes	13 (35.1)	9 (31.0)	4 (50.0)	
No	15 (40.5)	12 (41.4)	3 (37.5)	
NA	9 (24.3)	8 (27.6)	1 (12.5)	
**Alcohol** (%)				0.635
Yes	8 (21.6)	5 (17.2)	3 (37.5)	
No	18 (48.6)	14 (48.3)	4 (50.0)	
NA	11 (29.7)	10 (34.5)	1 (12.5)	

p-value: Wilcoxon test rank sum or Fisher’s exact test (two sided) was used for the comparison between the fusion burden high and low groups

NA: not available. NA was not included in statistical analysis

IQR: interquartile range

### DNA mutation analysis

DNA sequencing was performed on the Illumina platform after the libraries were constructed and purified. The clean reads were aligned to the human reference genome (Hg19, NCBI Build 37.5) using the Burrows-Wheeler Aligner (v. 0.7.17) [10] following the removal of low quality reads. Then, Picard toolkit (v. 2.1.0) [11] and Genome Analysis ToolKit (v. 3.7) [12] were used for making duplicates and for realignment, respectively. And Mutect 2 was utilized to identify single-nucleotide variants (SNVs) as well as small insertions and deletions (indels), while compound heterozygous mutations were merged by FreeBayes (v. 1.2.0) [13]. After ANNOVAR annotations [14], somatic mutations were picked out based on the following criteria: (i) located in intergenic or intronic regions; (ii) synonymous SNVs; (iii) allele frequency ≥ 0.002 in the Exome Aggregation Consortium (ExAC) and genomAD databases; (iv) allele frequency < 0.05 in the tumor samples and allele frequency < 0.01 in the plasma samples; (v) strand bias mutations in the reads; (vi) support reads< 5; and (vii) depth < 30. Subsequently, the identified tumor-related mutated genes were classified into ten signaling pathways and then subjected to Kyoto encyclopedia of genes and genomes (KEGG) pathway enrichment analyses.

### Calculation of TMB/bTMB and MATH

SNV mutations of all samples were filtered by using the following rules: (i) non-splicing sites or exonic regions; (ii) depth < 100X and allele frequency < 0.05; (iii) allele frequency ≥ 0.002 in the ExAC and genomAD databases; and (iv) strand bias mutations in the reads. And tumor mutational burden (TMB/bTMB) of the tumor tissue or blood was calculated based on the absolute mutation counts of the tumor samples against the mutation spots of the normal samples using the following formula: Absolute mutation counts*1000000/Total number of exonic bases. TMB/bTMB was measured in mutations per megabase (Mut/Mb).

With the Variant Allele Frequencies (VAF) determined by the ratio of alternate allele observations to the read depth at each site, we calculated the mutant-allele tumor heterogeneity (MATH) score including all somatic variants with a VAF ranging from 0.02 to 1 by the following formula: 100*median absolute deviation (MAD)/median of the VAF.

### Multiplex immunohistochemistry

An immune biomarker panel was utilized to quantitatively evaluate the following 11 distinct immune cell populations: PDL1+ cells, PD1+ cells, CD8+ cytotoxic T lymphocytes (CTLs), CD8 + PD1+ (Exhausted CTLs) vs CD8 + PD1- (Non-exhausted CTLs), CD68+ macrophages, CD68 + PDL1+ vs CD68 + PDL1-, CD57+ natural killer cells (NK), CD57 + PDL1+, and CD57 + PD1 + .

Multiplex immunohistochemistry (mIHC) was performed using an Opal™ 7-color IHC Kit (PerkinElmer Inc., Boston, MA, USA) following the manufacturer’s instructions. Antibodies used in this study included CD68 (1:500, Beijing Zhongshan Golden Bridge Biotechnology, ZM0060), CD8 (1:100, Beijing Zhongshan Golden Bridge Biotechnology, ZA0508), CD57 (1:100, Beijing Zhongshan Golden Bridge Biotechnology, ZM-0058), PD1 (1:50, Beijing Zhongshan Golden Bridge Biotechnology, ZM0381), and PDL1 (1:25, Roche Diagnostics, 740–4859). The slides were incubated with the primary antibodies, followed by incubation in 0.3% hydrogen peroxide solution for blocking endogenous peroxidase. The following fluorophores were used in the experiments: Opal 520, 540, 570, 620, 650 and 690. Nuclear counterstaining was conducted using DAPI. A Vectra 3.0.5 continuous spectrum imaging system (PerkinElmer Inc) and inForm 2.3.0 software (PerkinElmer Inc) were used respectively to acquire and analyze the images for tumor parenchyma (tumor), distant stroma and total regions.

### Statistical analysis

Survival analysis was performed to investigate survival differences between HNSCC patients with mutant genes (PIK3CA, TP53, PI3K pathway, p53 pathway and RTK_RAS pathway) and those with wild-type ones, and to examine the effects of factors such as TMB/bTMB, MATH and immune cell infiltration on survival. The best cutoff values were obtained by R language survival package analysis. Univariate survival analysis and multivariate analysis were carried out by using Log-rank test and Cox proportional hazards model, respectively. Log-rank test was also utilized to assess the association of the above variables with OS. Fisher’s Exact tests and Wilcoxon test were employed to detect the difference in the gene mutations, TMB/bTMB, MATH and immune cell infiltration between the response and non-response groups.

## Results

### Distribution of mutations in HNSCC

**Fig. 1:**
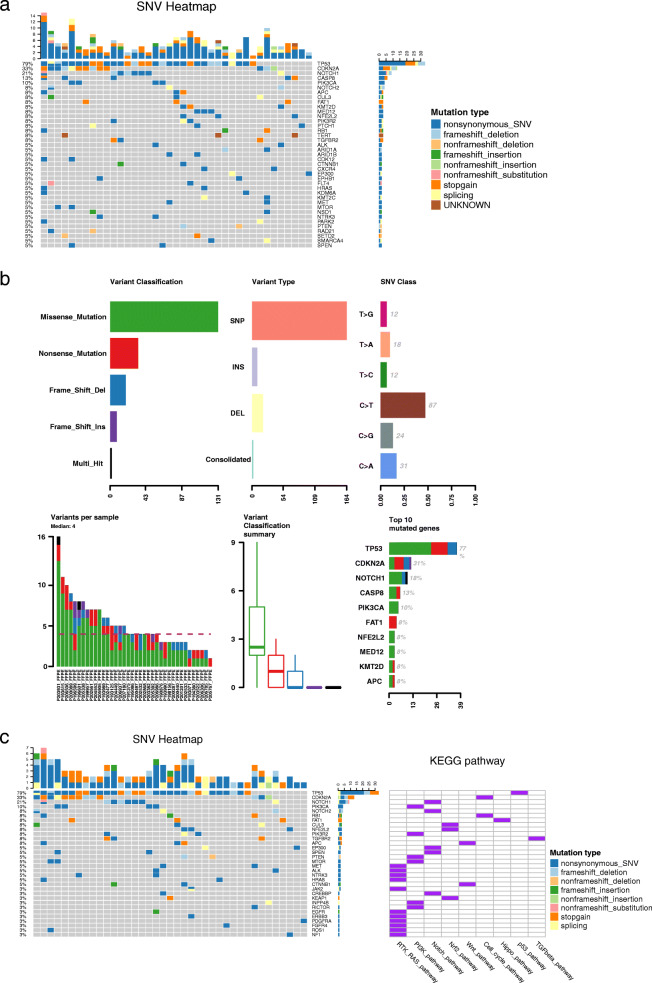
Genetic mutations in patients with advanced HNSCC after immunotherapy (*n* = 39). (**A**) The overall mutational landscape of patients; (**B**) Type analysis of mutated genes; (**C**) Enrichment pathway analysis of mutated genes

To analyze the distribution of mutations in HNSCC patients, we performed DNA mutation analysis. After excluding 5 samples with poor quality sequence data, the mutation patterns obtained from sequencing 39 tissue specimens were statistically compared. As shown in Fig. 1A-B, SNV and indel mutations occurred mainly in the samples, TP53 was the gene with the highest mutation frequency (79%) in the sample, followed by CDKN2A (33%), NOTCH1 (21%), CASP8 (13%), and PIK3CA (10%). In addition, we found that missense mutation and nonsense mutation were more frequent in HNSCC patients, with the highest frequency of base C to T mutations.

According to the classification of signaling pathways, tumor-related important genes can be assigned into the 10 major signaling pathways such as RTK-RAS, PI3K/Akt, beta-catenin/Wnt, and P53 pathways. In this study, no mutations were found in genes related to Myc pathway, and only 9 signaling pathways were annotated by the KEGG pathway enrichment analyses, with mutations mainly in the RTK-RAS pathway (Fig. 1C).

### Prognostic variables in HNSCC

**Fig. 2:**
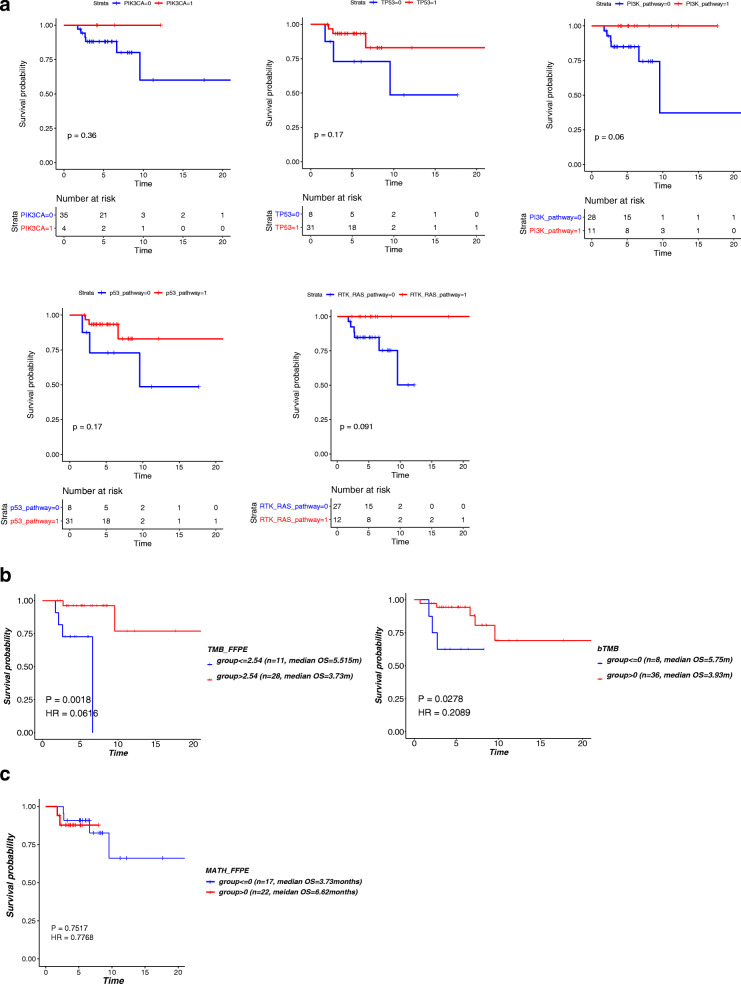
The relationship between gene/signal pathway mutations, TMB and MATH and OS. (**A**) Survival analysis based on mutations in different gene/signaling pathway, (**B**) Survival analysis based on TMB/bTMB values, (**C**) Survival analysis based on MATH values

As shown in Fig. 2A, compared with those harboring the wild type corresponding genes and pathways, patients with PIK3CA and TP53 gene mutations, PI3K pathway, p53 pathway, and RTK_RAS pathway mutations had a trend toward prolonged OS after PD1 treatment. However, no significant difference in OS was detected between the above two groups of patients (*n* = 39).

Previous studies have reported that TMB may be a potential biomarker for immunotherapy [15]. Therefore, we divided HNSCC patients into TMB/bTMB-high and TMB/bTMB-low groups based on the best cutoff value. A survival comparison of the two groups of patients found that patients with a high TMB/bTMB score who received PD1 treatment had significantly longer OS than those with a low TMB/bTMB score (*P* < 0.05) (Fig. 2B).

Next, we analyzed whether the value of MATH affects OS. For this purpose, the 39 patients were allocated into MATH-high group (MATH> 0, *n* = 22) and MATH-low group (MATH≤0, *n* = 17). As shown in Fig. 2C, there was no significant difference in OS between the above two groups, indicating that the MATH has no marked effect on OS of the HNSCC patients receiving PD1 immunotherapy (*P* > 0.05).

### Correlation of clinical characteristics and the proportion of immune cells with OS

**Fig. 3:**
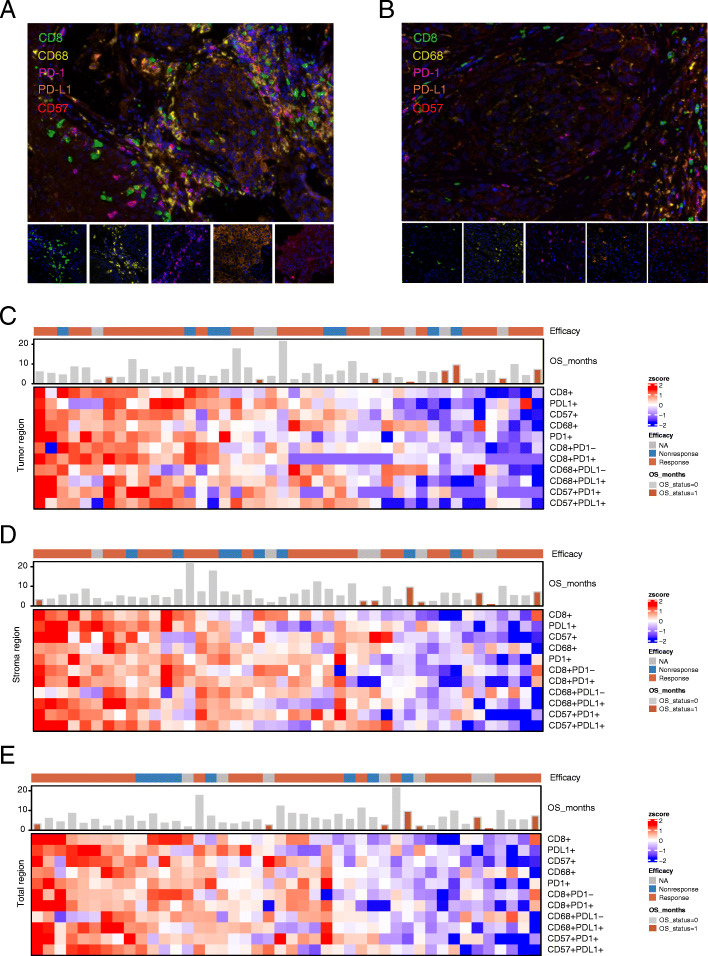
Immune landscape of HNSC patients treated with immunotherapy. The mIHC images represent (**A**) the group that responded to immunotherapy and (**B**) the group that did not respond to immunotherapy. The percentages of differentially expressed cells were log-transformed and z-score standardized. Heatmaps of immune cell infiltration in the (**C**) tumor region, (**D**) stroma region and (**E**) total region. The value in the lower left part of the diagonal in the table represents the correlation coefficient (spearman) of the expression percentage of each marker positive cell subgroup (r value): r > 0, positive correlation; r < 0, negative correlation. Specifically, a certain range of the r value indicates high correlation (0.8–1.0), strong correlation (0.6–0.8), moderate correlation (0.4–0.6), weak correlation (0.2–0.4), and very weak correlation or non-correlation (0.0–0.2). The r value was replaced by the pink circle in the upper right part of diagonal in the table. Pink and blue represent positive and negative correlations, respectively, while the asterisk at the top right represents the *P* value: * *P* < 0.05, ** *P* < 0.01, and *** *P* < 0.005

**Fig. 4:**
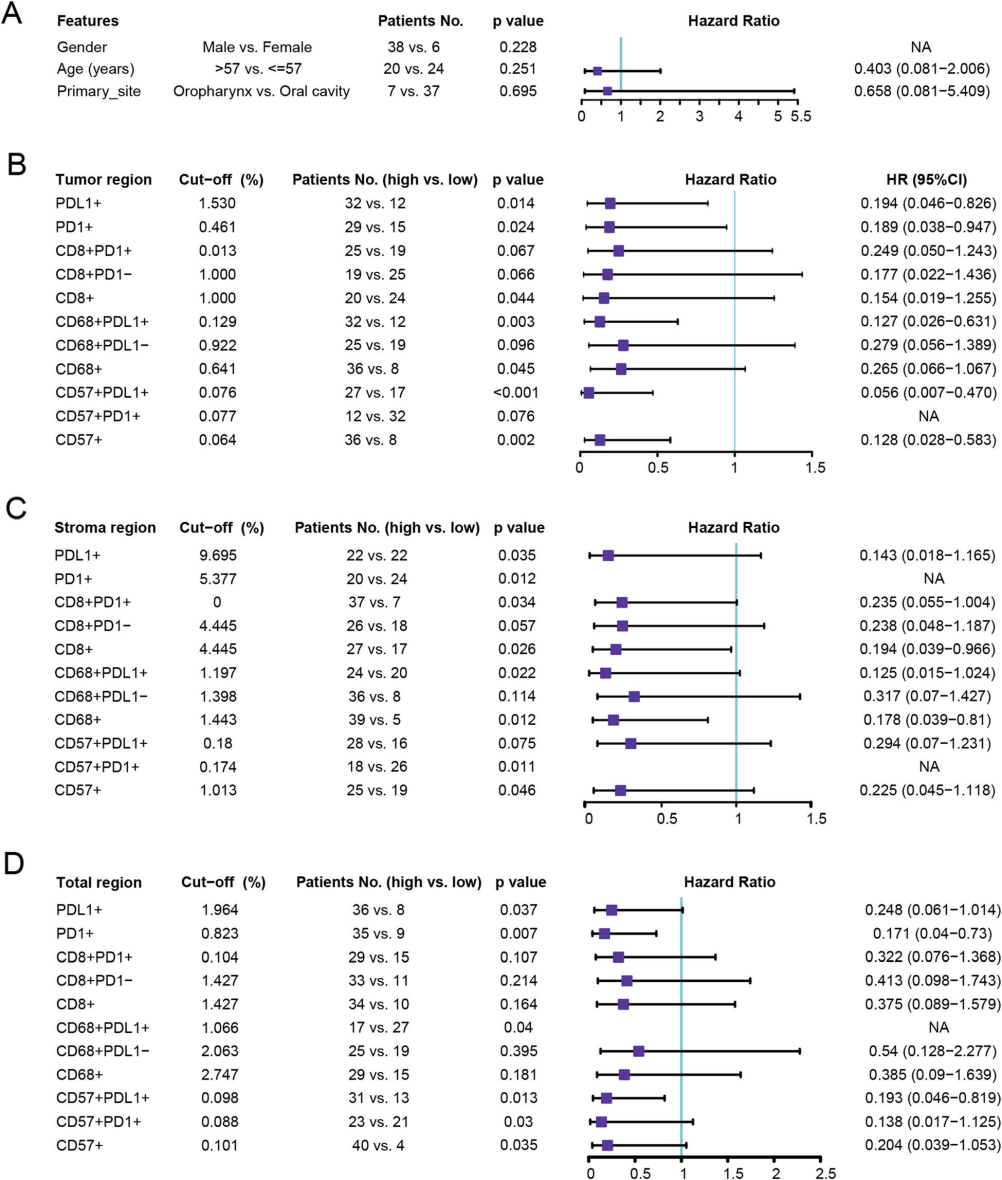
Univariate Cox regression analysis for overall survival

Then, we detected immunomarker-positive cells in the tumor, stroma and total area using Multiple IHC analysis (*n* = 37, Fig. 3A-B). In the above three areas, positive correlations were found between the immunomarker-positive cells, such as CD8+ and PD1+, CD57 + PDL1+ and CD68 + PDL1+, and CD57+ and CD68 + PDL1+ (Fig. 3C-E). Univariate Cox regression analysis revealed that patients’ gender, age and primary site were not significantly correlated with OS (*n* = 44, *P* > 0.05, Fig. 4A). In addition, multivariate survival analysis revealed no clinical features or tumor-associated inflammatory cells significantly associated with OS in both the stroma or total area (Figure S2A-B). Furthermore, patients with high expression of PD1+, PDL1+, CD8+, CD68+, CD68 + PDL1+, CD57+, CD57 + PDL1+, and CD57 + PD1+ had significantly prolonged OS after receiving PD1 treatment (Fig. 4B-D). Multivariate survival analysis showed that CD57 + PDL1+ in tumors was significantly associated with OS, but no clinical features and tumor-associated inflammatory cells significantly associated with OS were found in either the stroma or total area (Figure S2C).

### Identification of factors related to efficacy of PD1 treatment

SNV and indel mutations occurred predominantly in the tissues of patients undergoing PD1 efficacy evaluation, with relatively high frequencies of mutations in TP53 (82%), CDKN2A (33%), NOTCH1 (21%), CASP8 (12%) and PIK3CA (12%). Missense mutation was the most common mutation type, followed by nonsense mutation, while the most frequent base substitution pattern was C > T. Moreover, we identified RTK-RAS signaling pathway as one with the largest number of mutant genes in the tissue samples of 33 HNSCC patients. However, there was no significant difference between two groups (Figure S3).

We further analyzed the efficacy of PD1 treatment in the patients with respect to factors such as TMB/bTMB, MATH, and immune cell infiltration. As shown in Figure S4, no significant differences in TMB/bTMB and MATH scores were detected between the response and non-response groups. Meanwhile, the percentages of the infiltrating immune cells in tumor, stroma, and total regions showed no significant differences between the above two groups (Figure S5).

## Discussion

Evasion of immune surveillance has been identified as an important mechanism underlying the occurrence and development of HNSCC, highlighting the potential role of immunotherapy in improving the prognosis of the disease. As the most widely studied immunotherapy regimen for HNSCC patients, PD-1 pathway targeting has been shown to be effective in the treatment of advanced HNSCC [16, 17]. In this study, to identify biomarkers that may effectively predict the immunotherapy response of HNSCC, we examined the factors related to the efficacy and prognosis of immunotherapy by analyzing the relevant data of patients with advanced HNSCC receiving PD1 treatment.

HNSCC is characterized by frequent mutations that produce neoantigens. In 2015, the Cancer Genome Atlas (TCGA) published a comprehensive catalog of HNSCC somatic genome changes involving mutations in a large number of genes such as TP53, CDKN2A, NOTCH1, CASP8, and PIK3CA [18]. By analyzing the expression profiles of HNSCC patients downloaded from the TCGA database, Liu et al. identified six differentially expressed genes (DEGs) associated with OS in HNSCC patients (DKK1, HBEGF, RNASE7, TNFRSF12A, INHBA, and IPIK3R3), and developed a reliable DEG-based risk model [19]. It can better predict the ability of immunophenotype of HNSCC patients with potential prognostic value. By studying the immune cell infiltration, immune-related gene expression profiles and immune-related biological pathways in HNSCC patients, Zhu and colleagues found that combining anti-VEGF signaling pathway drugs with immunotherapy may be a new therapeutic direction [20]. Hanna et al. analyzed the mutations of HNSCC based on the anti-PD-1/L1 response, and found that the incidence of NOTCH1 mutations in the responders was significantly higher than that of non-responders [21]. In the present study, we showed that PD1 treatment led to a prolonged OS trend in patients with mutations in PIK3CA gene, TP53 gene, PI3K pathway, p53 pathway, and RTK_RAS pathway. Lyu et al. reported that TP53 mutations are associated with reduced immune markers, and multiple p53 and ras-mediated pathways are significantly related to HNSCC immunity [22]. Wild-type PIK3CA and TP53 are enriched in patients with HNSCC associated with immunosuppressive tumor microenvironment-related pathways and poor prognosis [23]. Inhibitors of PI3K/AKT/mTOR and NOTCH signaling pathways are promising molecularly targeted agents for the treatment of HNSCC [24]. However, statistical analysis revealed no significant impact of these mutations on OS and efficacy of PD1 treatment. This will require further validation of these mutated genes and pathways in subsequent studies.

Clinical trials have demonstrated that TMB is positively correlated with the efficacy and prognosis of immunotherapy in a variety of tumors. For example, compared with chemotherapy, immune checkpoint blockade therapy can significantly improve the median progression-free survival and the objective response rate in lung cancer patients with high TMB [25]. Another study in a clinical annotation cohort of HNSCC patients showed that TMB in patients who responded to PD-1/L1 treatment was significantly higher than that in those who did not, while TMB was markedly associated with improved prognosis [21]. Here, we observed that PD1 treatment led to a significantly prolonged OS in patients with higher TMB/bTMB. However, further analysis indicated that there was no significant difference in TMB between response group and non-response group, as well as in bTMB.

Accumulated evidence indicates that the tumor immune microenvironment (TIM) plays an important role in carcinogenesis as well as the regression or progression of HNSCC, implying prognostic relevance of the immune cell infiltration. For example, increased infiltration of CD3+, CD4+, CD8+, FoxP3+, CD20+ and CD56dim was detected in patients with oropharyngeal squamous cell carcinoma and was associated with improved overall survival [26–29]. In addition, patients with positive PD-L1 expression on immune cells had good disease-free survival (DFS) and OS. Notably, a training cohort analysis of 522 HNSCC cases from the Cancer Genome Atlas demonstrated that the enriched proinflammatory M1 macrophages signature and abundant tumor-infiltrating lymphocytes were associated with a good prognosis [30]. Consistent with these studies, we observed a significantly longer OS in HNSCC patients with highly infiltrated immune cells and high percentage of PD1+, PDL1+, CD8+, CD68+, CD68 + PDL1+, CD57+, CD57 + PDL1+ and CD57 + PD1+ cells. Further analysis with OS as the main endpoint revealed that the percentage of immunomarker-positive cells was significantly correlated with OS, whereas no marked correlation between the patient’s therapeutic efficacy and OS was identified. The above observations may explain why there was no difference in the expression of immunomarker-positive cells between the response and non-response groups.

It has been reported that HPV infection status can affect the infiltration of anti-tumor immune cell subsets in patients with HNSCC. In this study, the lack of clinical data on HPV infection status of the patients prevented us from analyzing the effect of HPV infection on the therapeutic efficacy, causing a certain impact on the results.

## Conclusion

In sum, HNSCC is a common malignant tumor characterized with high mutation rate, high mutation burden and high level of immune cell infiltration, and a subset of HNSCC patients respond to immunotherapy. The findings in this study suggested that high mutation burden and immune cell infiltration can improve the prognosis of HNSCC patients with immunotherapy, while there was no significant effect on the efficacy.

## Supplementary Information

**Additional file 1: Figure S1.** Diagram for the enrolled HNSC patients in the current study.

**Additional file 2: Figure S2.** Multivariate survival analysis results for clinical characteristics and enriched tumor-associated inflammatory cells in the (A) stroma and (B) tumor and (C) total region.

**Additional file 3: Figure S3.** Gene mutations in tissue samples of patients with advanced HNSCC receiving immunotherapy and efficacy evaluation. The patients were divided into the response group (*n* = 25) and non-response group (*n* = 8). SNV and indel mutations were detected in the patient’s tissues (A), and the identified mutations were summarized for the response group (B) and non-response group (C). Mutation diagram of the ten signaling pathways in tissue samples was illustrated (D).

**Additional file 4: Figure S4.** Statistical analysis of differences between the response and non-response groups. (A) TMB, (B) bTMB, (C) MATH values.

**Additional file 5: Figure S5.** Boxplot analysis of percentages of immunomarker positive cells between the response and non-response groups. (A) tumor region, (B) stroma region, (C) total region.

**Additional file 6: Table S1.** Clinical characteristics of enrolled HNSCC patients.

## Data Availability

The datasets used and/or analyzed during the current study are available from the corresponding author on reasonable request.

## Abbreviations

HNSCC: Head and neck squamous cell carcinoma
OS: Overall survival
ExAC: Exome Aggregation Consortium
KEGG: Kyoto encyclopedia of genes and genomes
TMB/bTMB: Tumor mutational burden
Mut/Mb: Mutations per megabase
VAF: Variant Allele Frequencies
MATH: Mutant-allele tumor heterogeneity
MAD: Median absolute deviation
mIHC: Multiplex immunohistochemistry
TCGA: The Cancer Genome Atlas
TIM: Tumor immune microenvironment
DFS: Disease-free survival
