# Supplementary figures and images for: Identification of factors related to immunotherapy efficacy and prognosis in patients with advanced head and neck squamous cell carcinoma
Source: Diagn Pathol. 2021 Nov 25;16:110. doi: 10.1186/s13000-021-01147-7 (PMC8620526; doi:10.1186/s13000-021-01147-7)

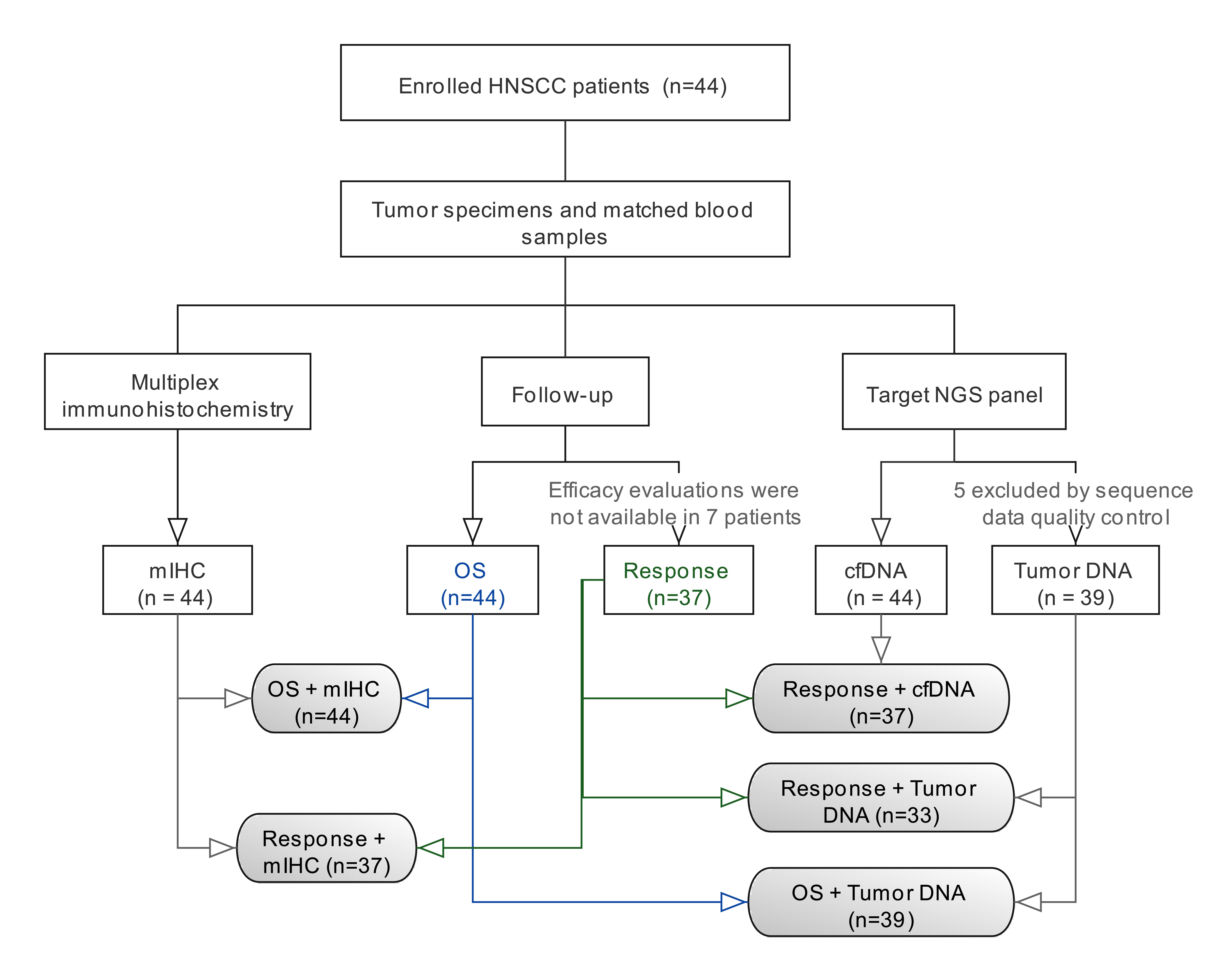

Supplement: Supplementary file 1 — Additional file 1: Figure S1. Diagram for the enrolled HNSC patients in the current study. [file 13000_2021_1147_MOESM1_ESM.jpg]

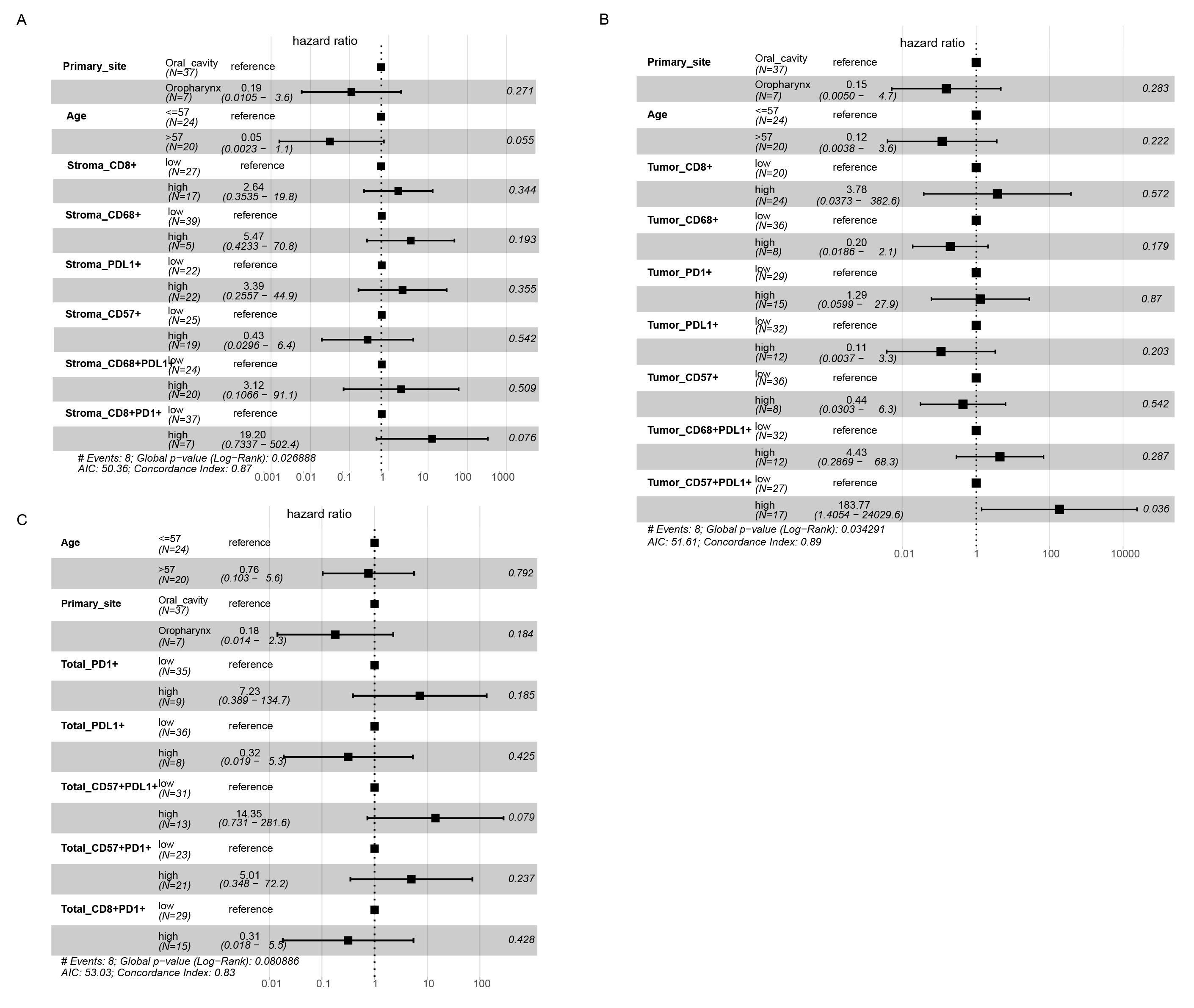

Supplement: Supplementary file 2 — Additional file 2: Figure S2. Multivariate survival analysis results for clinical characteristics and enriched tumor-associated inflammatory cells in the (A) stroma and (B) tumor and (C) total region. [file 13000_2021_1147_MOESM2_ESM.tif]

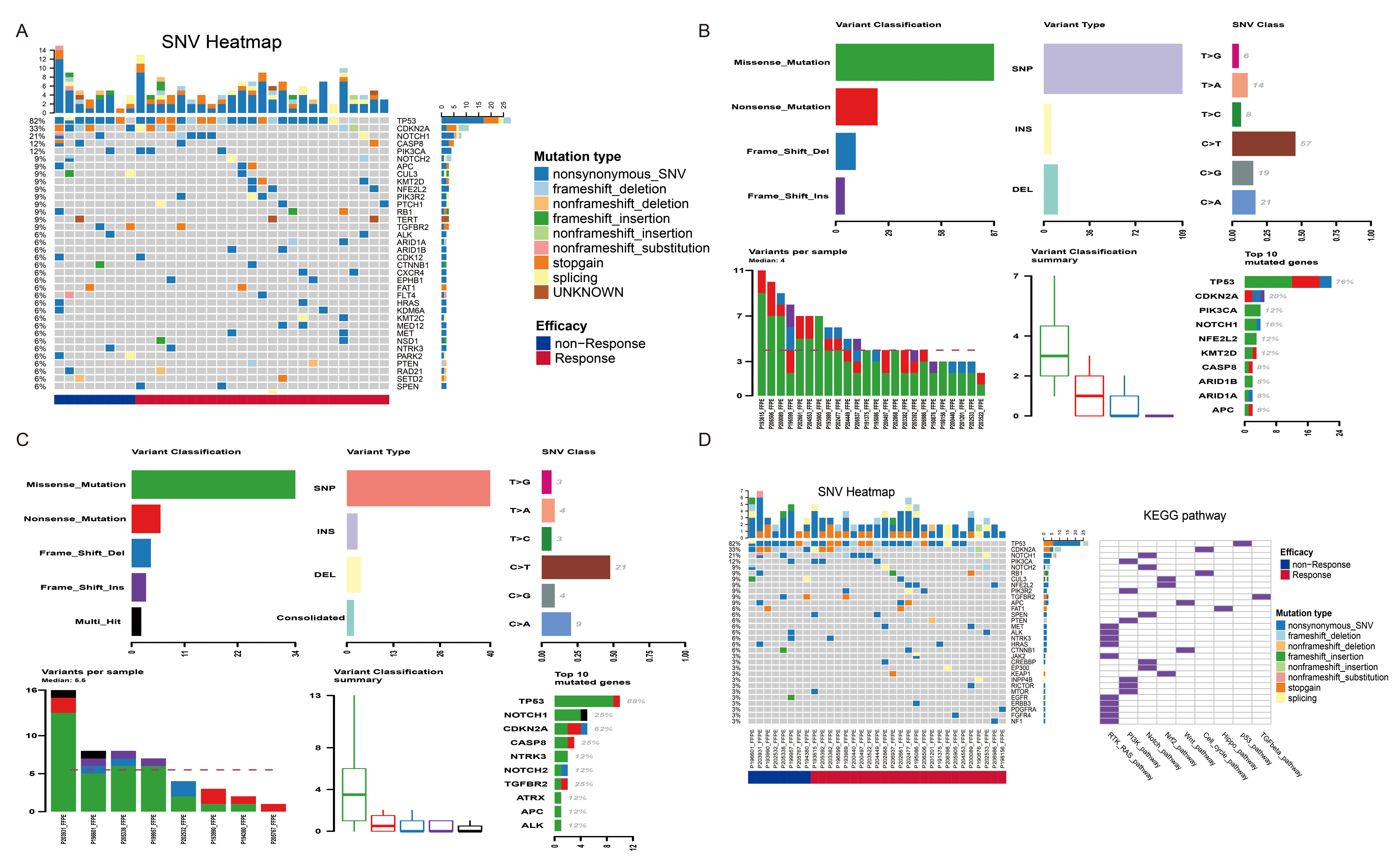

Supplement: Supplementary file 3 — Additional file 3: Figure S3. Gene mutations in tissue samples of patients with advanced HNSCC receiving immunotherapy and efficacy evaluation. The patients were divided into the response group (n = 25) and non-response group (n = 8). SNV and indel mutations were detected in the patient’s tissues (A), and the identified mutations were summarized for the response group (B) and non-response group (C). Mutation diagram of the ten signaling pathways in tissue samples was illustrated (D). [file 13000_2021_1147_MOESM3_ESM.jpg]

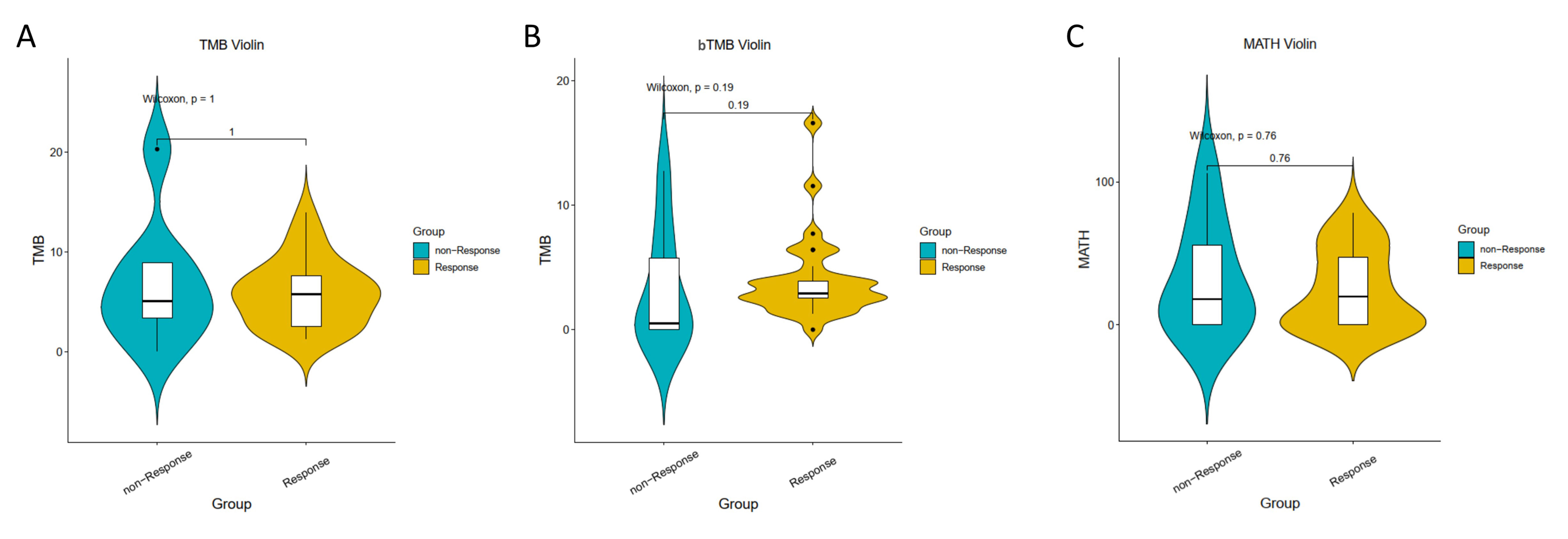

Supplement: Supplementary file 4 — Additional file 4: Figure S4. Statistical analysis of differences between the response and non-response groups. (A) TMB, (B) bTMB, (C) MATH values. [file 13000_2021_1147_MOESM4_ESM.jpg]

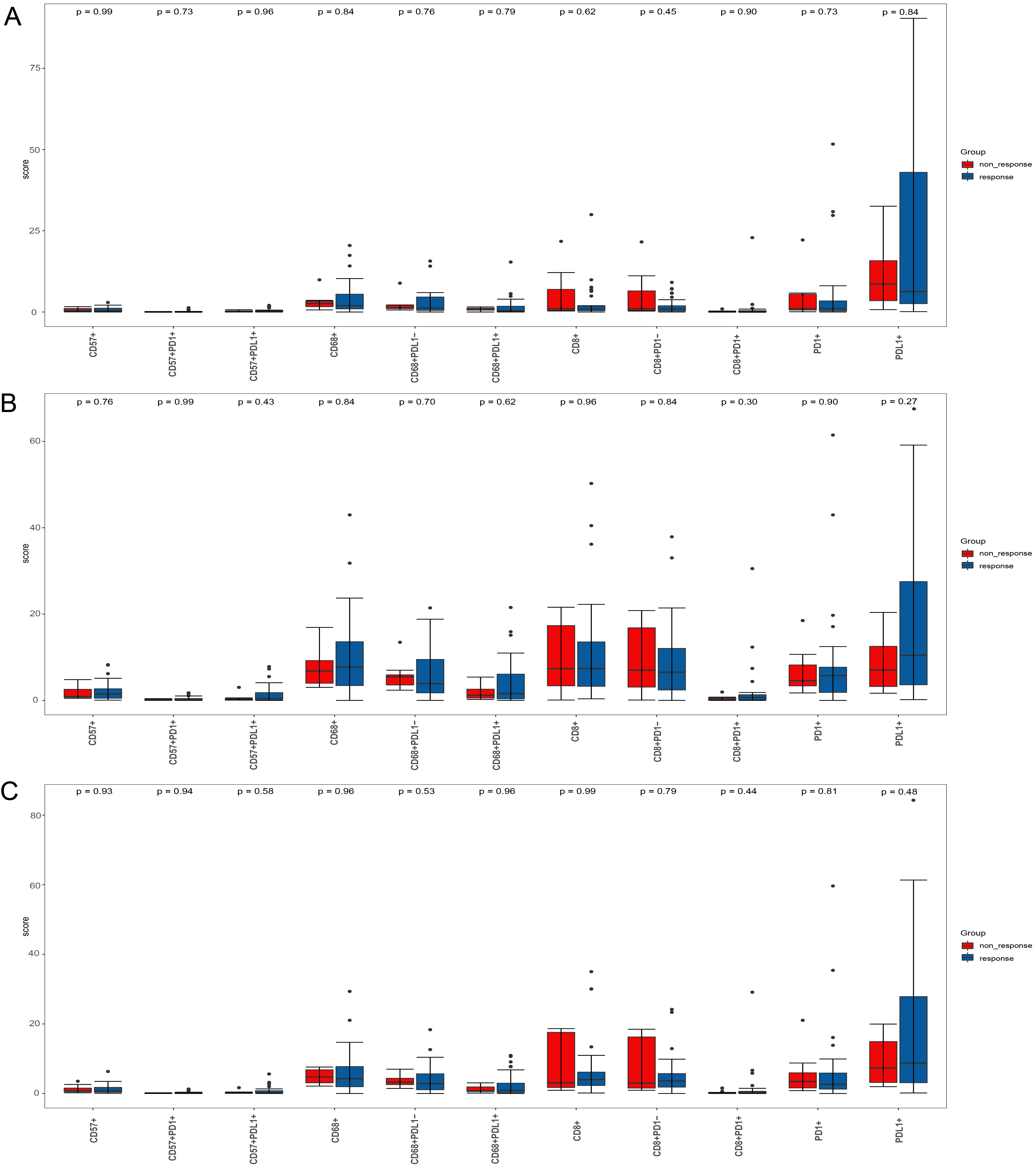

Supplement: Supplementary file 5 — Additional file 5: Figure S5. Boxplot analysis of percentages of immunomarker positive cells between the response and non-response groups. (A) tumor region, (B) stroma region, (C) total region. [file 13000_2021_1147_MOESM5_ESM.jpg]
