# Supplementary material for: Identification of factors related to immunotherapy efficacy and prognosis in patients with advanced head and neck squamous cell carcinoma
Source: Diagn Pathol. 2021 Nov 25;16:110. doi: 10.1186/s13000-021-01147-7 (PMC8620526; doi:10.1186/s13000-021-01147-7)
Supplement: Supplementary file 6 — Additional file 6: Table S1. Clinical characteristics of enrolled HNSCC patients. [file 13000_2021_1147_MOESM6_ESM.docx]

Table S1. Clinical characteristics of enrolled HNSCC patients.

| **Characteristic** | | **Overall (n=44)** |
| --- | --- | --- |
| **Gender** (%) | Female | 6 (13.6) |
|  | Male | 38 (86.4) |
| **Age** (years) | median | 57.0 |
|  | IQR | 50.8-64.5 |
| **Primary site** (%) | Oral cavity | 37 (84.1) |
|  | Oropharynx | 7 (15.9) |
| **HPV infection status** (%) | Negative | 7 (15.9) |
|  | NA | 37 (84.1) |
| **pStage** (%) | IVa | 30 (68.2) |
|  | IVb | 2 (4.5) |
|  | IVc | 9 (20.5) |
|  | NA | 3 (6.8) |
| **T stage** (%) | T1 | 2 (4.5) |
|  | T2 | 4 (9.1) |
|  | T4 | 23 (52.3) |
|  | NA | 15 (34.1) |
| **N stage** (%) | N1 | 4 (9.1) |
|  | N2 | 24 (54.5) |
|  | N3 | 1 (2.3) |
|  | NA | 15 (34.1) |
| **M stage** (%) | M1 | 9 (20.5) |
|  | NA | 35 (79.5) |
| **Tobacco** (%) | Yes | 13 (29.5) |
|  | No | 22 (50.0) |
|  | NA | 9 (20.5) |
| **Alcohol** (%) | Yes | 8 (18.2) |
|  | No | 25 (56.8) |
|  | NA | 11 (25.0) |

NA: not available; IQR: interquartile range
